# Application of RP-18 TLC Retention Data to the Prediction of the Transdermal Absorption of Drugs

**DOI:** 10.3390/ph14020147

**Published:** 2021-02-12

**Authors:** Anna W. Sobańska, Jeremy Robertson, Elżbieta Brzezińska

**Affiliations:** 1 Department of Analytical Chemistry, Faculty of Pharmacy, Medical University of Lodz, ul. Muszyńskiego 1, 90-151 Łódź, Poland; elzbieta.brzezinska@umed.lodz.pl; 2 Chemistry Research Laboratory, Department of Chemistry, University of Oxford, Mansfield Road, Oxford OX1 3TA, UK; jeremy.robertson@chem.ox.ac.uk

**Keywords:** skin permeation, thin layer chromatography, computational descriptors

## Abstract

Several chromatographic parameters (***R_M_*^0^** and ***S*** obtained from RP-18 TLC with methanol—pH 7.4 phosphate buffer mobile phases by extrapolation to zero concentration of methanol; ***R_f_*** and ***R_M_*** obtained from RP-18 TLC with acetonitrile—pH 7.4 phosphate buffer 70:30 *v*/*v* as a mobile phase) and calculated molecular descriptors (molecular weight—***M_W_***; molar volume—***V_M_***; polar surface area—***PSA***; total count of nitrogen and oxygen atoms—(***N+O***); H-bond donor count—***HD***; H-bond acceptor count—***HA***; distribution coefficient—log ***D***; total energy—***E_T_***; binding energy—***E_b_***; hydration energy—***E_h_***; energy of the highest occupied molecular orbital—***E_HOMO_***; energy of the lowest unoccupied orbital—***E_LUMO_***; electronic energy—***E_e_***; surface area—***S_a_***; octanol-water partition coefficient—log ***P***; dipole moment—***DM***; refractivity—***R***, polarizability—***α***) and their combinations (***R_f_***/***PSA***, ***R_M_***/***M_W_***, ***R_M_***/***V_M_***) were tested in order to generate useful models of solutes’ skin permeability coefficient log ***K_p_***. It was established that neither ***R_M_*^0^** nor ***S*** obtained in the conditions used in this study is a good predictor of the skin permeability coefficient. The chromatographic parameters ***R_f_*** and ***R_f_/PSA*** were also unsuitable for this purpose. A simple and potentially useful, purely computational model based on (***N+O***), log ***D*** and ***HD*** as independent variables and accounting for *ca.* 83% of total variability was obtained. The evaluation of parameters derived from ***R_M_*** (***R_M_***, ***R_M_***/***M_W_***, ***R_M_***/***V_M_***) as independent variables in log ***K_p_*** models proved that ***R_M_***/***V_M_*** is the most suitable descriptor belonging to this group. In a search for a reliable log ***K_p_*** model based on this descriptor two possibilities were considered: a relatively simple model based on 5 independent variables: (***N+O***), log ***D***, ***R_M_***/***V_M_***, ***E_T_*** and ***E_h_*** and a more complex one, involving also ***E_b_***, ***M_W_*** and ***PSA***.

## 1. Introduction

The skin is the heaviest organ in the human body. Its average surface area is ca. 2 m2 and it accounts for about 1/_10_ of the total bodyweight [1]. The skin provides a selective barrier, allowing transdermal delivery of drugs and providing protection against harmful chemicals. Topically applied drugs and other compounds enter the body via either the transepidermal pathway (diffusion across the skin layers) or the appendageal pathway (through hair follicles or sweat ducts), the latter being considered significantly less important [1]. In the transepidermal pathway the molecule permeates the skin either transcellularly or intercellularly and the preferred route depends on the solute’s molecular properties—small hydrophilic molecules prefer the transcellular route and lipophilic ones favor the opposite [1]. The rate of drug permeation through skin is expressed as the flux (***J***)—the amount of substance permeated per unit area and unit time. The flux depends on the permeabilityof the skin to the permeant (***K_p_***) and the gradient of permeant concentration across the skin(Δ***c***): ***J*** = ***K_p_***∙Δ***c.***

For passive diffusion, the permeability coefficient ***K_p_*** depends on the partition coefficient ***P***, the diffusion coefficient ***D*** and the diffusional pathlength *h*: ***K_p_*** = ***P***∙***D***/***h***.

The ability of compounds to cross the skin barrier is of great interest to pharmaceutical and medicinal chemists because the transdermal delivery of drugs is an effective alternative to systemic delivery. This ability is also important in the context of environmental toxicology, because many harmful substances enter the body through skin. Transdermal permeation of drugs may be studied using many methods, including in vitro experiments on excised human skin [2] but there are many problems connected with this methodology. Aside from ethical considerations, the compound’s skin permeability may differ significantly between individuals, depending on age and race and even between skin samples taken from the same individual. Percutaneous absorption is therefore studied on other, more convenient, models, including pig, rabbit, rat, mouse or shed snake skin, cultured human skin cells, or synthetic membranes [2,3]. However, such models require much tedious experimental work, so alternative solutions have been sought. Skin permeation depends on some readily obtained physicochemical parameters of a molecule, including the well-established predictor of lipophilicity and biological activity of compounds, the partition coefficient between aqueous and organic layers log ***P_ow_*** [4]. However, it was demonstrated that log ***P_ow_*** cannot be applied as a single measure of log ***K_p_*** across a very wide range of chemical families, so molecular weight or volume were incorporated as additional descriptors [5,6]. Further research supported a relationship between ***K_p_*** and hydrogen bond donor and acceptor activity (***H_d_*** and ***H_a_***, respectively) [7] and a melting point ***M_Pt_*** [8]. Other authors stressed the importance of solvation free energy [9]. Log ***K_p_*** was also correlated with Abraham’s solute descriptors [10,11,12]. Following QSAR studies on skin permeation, models involving log ***P_ow_*** and ***M_w_*** along with further descriptors were developed [13,14,15]. Different computational skin permeability models have been reviewed and compared [15,16,17,18,19,20,21,22], and the most interesting equations discussed there are presented in Table 1.

More recent developments in the field of in silico evaluation of skin permeability include the model proposed by Chenet et al., [24], in which predictions of skin permeability may be based on certain molecular properties and topological descriptors.

The equation proposed by Potts is among the most accurate and the most frequently cited of the computational models of skin permeability coefficient [21,22], although the model developed by Mitragotri [25] is also interesting [21]—supposedly giving even more reliable predictions—but the model by Potts has the benefit of simplicity.

Chromatography is a powerful technique for acquiring measurements of physicochemical and biological properties of solutes, including the ability of compounds to cross different biological barriers [26,27,28]. It is superior to techniques based on excised human skin, animal experiments or even cell cultures because it is more reproducible and it usually involves commercially available chromatographic supports. The chromatographic techniques used to predict the skin permeability of solutes include normal- and reversed-phase thin layer chromatography [29,30], immobilized artificial membrane (IAM) column chromatography [31,32,33,34], RP-18 column chromatography [33,34,35], column chromatography on a novel stationary phase based on immobilized keratin [36], biopartitioning micellar chromatography (BMC) [37,38,39], micellar electrokinetic chromatography [34], and liposome electrokinetic chromatography [40]. Available models of skin permeability parameters, based on liquid chromatographic descriptors, are presented in Table 2.

From the equations presented in Table 2, it can be concluded that skin permeability coefficient is connected with chromatographic parameters (log ***k*** or ***R_M_*^0^** for column and thin layer chromatography, respectively) *via* linear or reverse parabolic relationships. Chromatographic retention parameters are used either as sole skin permeability predictors, or they are combined with additional descriptors (log ***P_ow_***, ***V***, ***M_w_*** or ***M_Pt_***). 

The objective of this study was to examine the relationships between the skin permeability coefficient log ***K_p_*** and different, RP-18 chromatography-derived descriptors and other physicochemical parameters for a large group of structurally unrelated compounds, mainly drugs.

## 2. Results and Discussion

### 2.1. General Considerations

The majority of compounds analyzed in this study are drugs, administered either orally or in the form of injections, and whose transdermal delivery, in certain cases, is of interest. On the other hand, some topically applied substances (e.g., cosmetic preservatives) may exhibit the unwanted ability to cross the skin barrier. The skin permeability coefficient (***K_p_***) is an important parameter that helps in the assessment of compounds’ epidermal permeability; however, the experimentally determined values of ***K_p_*** are available for only a few drugs, within the studied group. For this reason it was decided that the models of skin permeability involving thin layer chromatographic and calculated descriptors should be generated and validated using ***K_p_*** values obtained *in silico* with the EpiSuite software (DERMWIN v. 2 module) (log ***K_p_***^EPI^), recommended by the US Environmental Protection Agency [41] and, at a final stage of these investigations, tested on a sub-group of analyzed solutes whose experimental log ***K_p_*** values could be found (log ***K_p_***^exp^). The estimation methodology used by DERMWIN is based on the Equation (2), related to the Potts model:

log ***K_p_*** (cm/h) = −2.80 + 0.66 log ***P_ow_*** − 0.0056***M_W_*** (R^2^ = 0.66) (1)

The values of log ***K_p_*^EPI^** obtained using DERMWIN are given in Table 3.

### 2.2. Thin Layer Chromatographic Parameters—Extrapolation Methodology

Reversed-phase thin layer chromatography has been used to predict physicochemical properties and bioavailability of compounds for many years [26] and the chromatographic parameter considered in these investigations is usually the ***R_M_*** value defined by Bate-Smith and Westall (Equation (2)) [42]:

***R_M_*** = log (1/***R_f_*** − 1) (2)

The partitioning between chromatographic supports and an aqueous mobile phase resembles that between biomembranes and the aqueous phase. The most common approach to obtain the chromatographic retention parameters for water as a mobile phase is by using a series of chromatographic experiments with mobile phases containing different concentrations ***φ*** of a water-miscible solvent (organic modifier). Plots of ***R_M_*** vs. ***φ*** are extrapolated to zero concentration of the modifier to furnish ***R_M_*^0^** and the most common method to do so is by using the linear Soczewiński-Wachmeister Equation (3) [43].

R_M_ = R_M_^0^ + S∙φ (3)

Apart from the ***R_M_*^0^** value, other useful chromatographic descriptors derived from the linear extrapolation of ***R_M_*** to purely aqueous conditions are the slope ***S*** and ***C*_0_** = −***R_M_*^0^/*S***.

**Figure 1:**
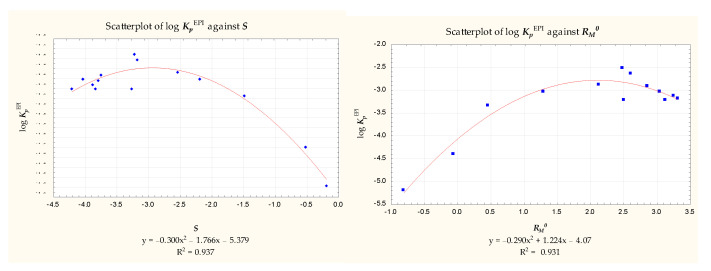
Scatterplots of log ***K_p_***^EPI^ vs. ***S*** and ***R_M_*^0^** (*n* = 14).

The compounds **1** to **22** and **62** were chromatographed on the RP-18 stationary phase using methanol—pH 7.4 buffer mobile phases as described in Section 3. The values of ***R_M_*** were calculated and plotted against the organic modifier concentration ***φ***. The chromatographic parameters ***R_M_*^0^** and ***S*** obtained according to Equation (3) (Table 4) were correlated with the log ***K_p_***^EPI^ values presented in Table 3, and the resulting correlations were found to be unsatisfactory. Neither ***R_M_*^0^** nor ***S*** correlated with log ***K_p_***^EPI^ for the whole group of 23 compounds. 14 compounds (**2**, **3**, **5**, **6**, **8**, **9**, **10**, **11**, **16**, **17**, **18**, **20**, **21**, **62**) gave reverse parabolic relationships with ***R_M_*^0^** and ***S*** (Figure 1), but the remaining solutes did not fit any reasonable pattern.

From this, it was evident that ***R_M_*^0^** and ***S*** obtained in the chromatographic conditions described in Section 3 could not be used as sole predictors of log ***K_p_***. Attempts to generate a multivariate relationship between log ***K_p_*** and the calculated physicochemical parameters, involving ***R_M_*^0^** or ***S***, failed and a purely computational model (4) was obtained by forward stepwise regression:

log ***K_p_*** = 7.340 (±2.265) + 0.00018 (±0.00376) ***PSA*** + 0.803 (±0.096) log ***D*** + 0.980 (±0.235) ***E_HOMO_*** − 0.959 (±0.184) ***E_LUMO_*** + 0.049 (±0.018) ***E_h_*** − 0.116 (±0.019) ***α*** (n = 23, R^2^ = 0.948, R^2^_adj._ = 0.928, F = 48.367, *p* < 0.00000, s_e_ = 0.316) (4)

**Figure 2:**
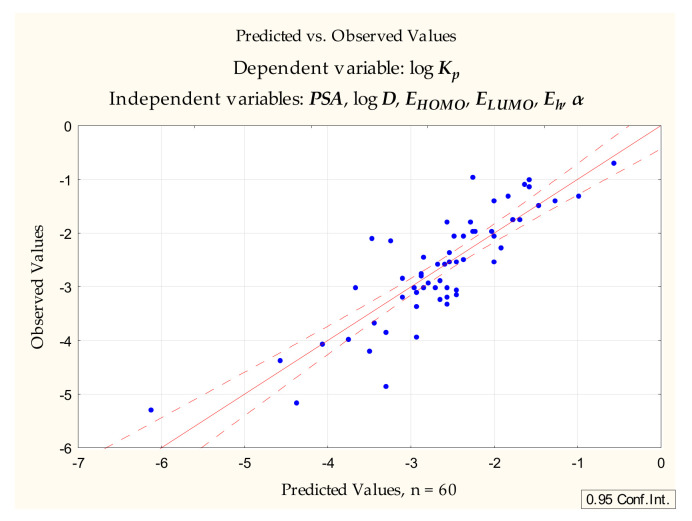
Equation (5)—predicted vs. observed values.

The same set of dependent variables was applied to compounds **1** to **60** to furnish the Equation (5) (Figure 2):

log ***K_p_*** = −1.524 (±1.386) − 0.0176 (±0.0042) ***PSA*** + 0.241 (±0.058) log ***D*** + 0.0149 (±0.140) ***E_HOMO_*** + 0.0472 (±0.189) ***E_LUMO_*** + 0.0311 (±0.0215) ***E_h_*** − 0.0113 (±0.0110) ***α*** (*n* = 60, R^2^ = 0.731, R^2^_adj._ = 0.700, F = 23.994, *p* < 0.00000, s_e_ = 0.572) (5)

The Equations (4) and (5), based on the set of purely computational variables ***PSA***, log ***D***, ***E_HOMO_***, ***E_LUMO_***, ***E_h_*** and ***α***, are relatively simple and logical, since they involve some properties responsible for drug absorption (lipophilicity, polar surface area and polarizability) [44,45]. However, apart from poor statistics, the differences between the parameters of the Equations (4) and (5) suggest that they lack the required universality with respect to larger groups of structurally unrelated compounds.

### 2.3. Single Chromatographic Run Retention Parameters—R_f_

The extrapolation method, although commonly used and recognized, has certain drawbacks. Several chromatographic experiments are required and the extrapolated ***R_M_*^0^** values depend on an organic modifier and its concentration range used to generate ***R_M_*** = *f*(***φ***) plots. Some studies, therefore, use the single chromatographic run approach in which the ***R_f_*** and ***R_M_*** values are collected using a single concentration of an organic modifier in a mobile phase [46]. The single chromatographic run approach was used to predict the lipophilicity of selected cosmetic raw materials [47] and, in separate work, yielded two chromatographic descriptors, ***R_f_*** and ***R_f_*/*PSA***, used to study the blood-brain barrier permeation of solutes [48,49,50]. Apart from providing reliable lipophilicity descriptors, this methodology has additional advantages: it requires considerably less experimental work than any extrapolation or interpolation method, and none of the additional factors mentioned earlier (such as the modifier concentration range or the type of extrapolation curve) need to be taken into consideration.

**Figure 3:**
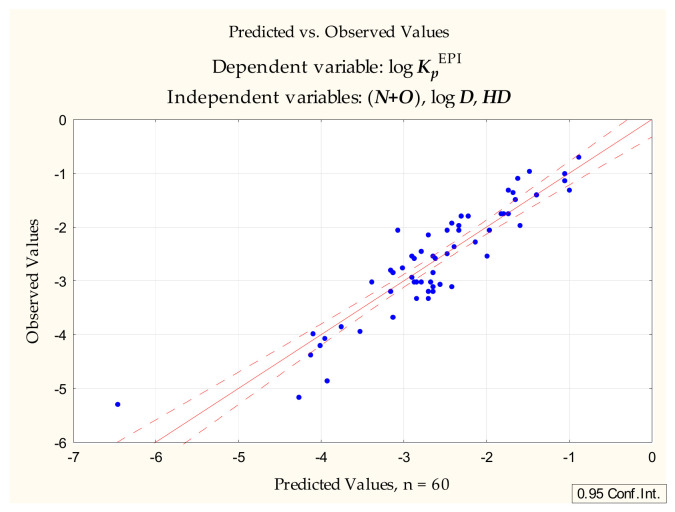
Equation (6)—predicted vs. observed values.

Following the unsuccessful attempts to generate useful and universal models of the skin permeability coefficient using thin layer chromatographic retention parameters obtained by extrapolation (***R_M_*^0^**, ***S***), attention turned to the single RP-18 chromatographic run approach developed in the earlier study of blood-brain barrier permeation [48,49,50]. The models presented in those studies involve ***R_f_*** (collected as described in Section 3) and a novel parameter derived from it: ***R_f_***/***PSA***. The latter (combined with ***R_f_*** and a number of calculated physicochemical parameters) is a particularly good predictor of the blood-brain barrier permeability (expressed as steady state blood-brain partition ratio, log ***BB***). In the case of the skin permeability coefficient, however, this parameter (and ***R_f_*** itself) failed to be selected in the course of the forward stepwise regression. The most interesting model (6) (Figure 3) generated at this stage of our investigations did not contain any chromatographic parameters and was as follows:

log ***K_p_*** = −1.390 (±0.181) − 0.352 (±0.034) (***N+O***) + 0.155 (±0.037) log ***D*** − 0.229 (±0.062) ***HD*** (n = 60, R^2^ = 0.833, R^2^_adj._ = 0.824, F = 92.270, *p* < 0.0000, s_e_ = 0.438) (6)

This model accounts for over 83% of total variability and contains the independent variables introduced in the following order: (***N+O***), log ***D***, ***HD***. The variables in the equation are strongly related to the conditions of good oral availability [44,45] and the ability to cross the blood-brain barrier [48,49,50,51]. The equation obtained at the very first stage of this regression, containing (***N+O***) as a sole independent variable, accounts for as much as 70% of total variability. The coefficients for (***N+O***) and ***HD*** are negative, which (as already observed, e.g., by Lien and Gaot [23]) suggests that excessive hydrogen bonding is an obstacle to epidermal permeability. 

At this point the group of 60 studied compounds was divided into two subsets: a training set (**1** to **40**) and a test set (**41** to **60**).Equation (7) generated for the training set, and containing the same independent variables as Equation (6), was as follows:

log ***K_p_*** = −1.561 (±0.248) − 0.319 (±0.041) (***N+O***) + 0.177 (±0.053) log ***D***− 0.256 (±0.072) ***HD*** (*n* = 40, R^2^ = 0.819, R^2^_adj._ = 0.804, F = 54.356, *p* < 0.00000, s_e_ = 0.456) (7)

The values of log ***K_p_*** were calculated for compounds **41** to **60** according to Equation (7) and plotted against the reference log ***K_p_***^EPI^ values presented in Table 3. The linear relationship between these two groups of log ***K_p_*** values improved when salicylic acid (**60**), whose ***K_p_***^EPI^ value seems to be overestimated compared to ***K_p_***^exp^ (due to the molecule’s combined polarity and acidic properties, **60** is not a very good skin permeant), was removed as an outlier (R^2^ = 0.83 and 0.89, respectively).

The model (6) was also tested on a subgroup of 9 compounds, analyzed in this study, whose log ***K_p_***^exp^ values were available (**12**, **16**, **21**, **40**, **56**, **60**, **61**, **62**, **63**). The resulting dependence is linear, with R^2^ = 0.92, which is a much better result than that obtained for the relationship between log ***K_p_***^exp^ and log ***K_p_***^EPI^ (for *n* = 9, R^2^ = 0.40).

### 2.4. Single Chromatographic Run Retention Parameters—**R_M_**

**Figure 4:**
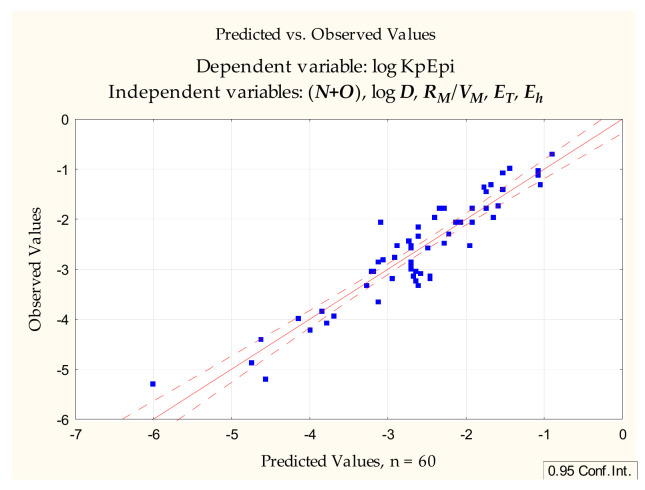
Equation (8)—predicted vs. observed values.

With the promising equations (6) and (7) in hand, attention turned to the possibility of using ***R_M_*** values calculated from ***R_f_*** obtained according to Section 3. Apart from ***R_M_*** itself, other TLC-derived variables were tested: ***R_M_***/***V_M_*** and ***R_M_***/***M_W_***. The first model (8) involving ***R_M_***–derived parameters, generated by forward stepwise multiple regression, was as follows (Figure 4):

log ***K_p_*** = −1.651 (±0.185) − 0.370 (±0.034) (***N+O***) + 0.130 (±0.034) log ***D*** + 90.44 (±43.04) (***R_M_***/***V_M_***) − 0.0000058 (±0.0000019) ***E_T_*** +0.0293 (±0.014) ***E_h_*** (*n* = 60, R^2^ = 0.868, R^2^_adj._ = 0.855, F = 70.869, *p* < 0.0000, s_e_ = 0.397) (8)

In Equation (8) the independent variables were introduced in the following order: (***N+O***), log ***D***, ***R_M_***/***V_M_***, ***E_T_***, ***E_h_***. The difference between this model and Equation (6) was not very significant; ***HD*** was replaced with ***R_M_***/***V_M_***, and two more independent variables, responsible for a very small improvement in statistics, were introduced (***E_T_***, ***E_h_***), but (***N+O***) and log ***D*** still accounted for the greatest percentage of variability.

The group of 60 studied compounds was, as before, divided into a training set (**1** to **40**) and a test set (**41** to **60**). Equation (9), generated for the training set and containing the same independent variables as Equation (8), was as follows:

log ***K_p_*** = −1.602 (±0.296) − 0.346 (±0.072) (***N+O***) + 0.156 (±0.067) log ***D*** + 108.57 (±51.25) (***R_M_***/***V_M_***) − 0.0000024 (±0.0000058) ***E_T_*** + 0.0254 (±0.0187) ***E_h_*** (n = 40, R^2^ = 0.823, R^2^_adj._ = 0.797, F = 31.654, *p* < 0.00000, s_e_ = 0.464) (9)

Values of log ***K_p_*** were calculated for compounds **41** to **60** according to Equation (9) and were plotted against the reference log ***K_p_***^EPI^ values. Just as in the case of Equation (7), the linear relationship between these two groups of log ***K_p_*** values improved when salicylic acid (**60**) was removed as an outlier (R^2^ = 0.87 and 0.92, respectively).

The model (8) was also tested on a subgroup of nine compounds, analyzed in this study, whose log ***K_p_***^exp^ values were available (**12**, **16**, **21**, **40**, **56**, **60**, **61**, **62**, **63**). The resulting relationship was linear, with R^2^ = 0.80, which is a much better result than that obtained for the relationship between log ***K_p_***^exp^ and log ***K_p_***^EPI^ (for *n* = 9, R^2^ = 0.40), but not as good as in the case of the Equation (6).

**Figure 5:**
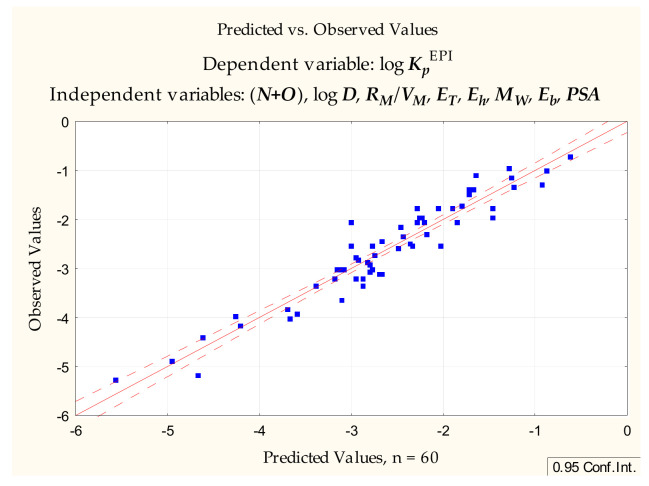
Equation (10), predicted vs. observed values.

Equations (8) and (9) account for 87 and 82% of total variability, respectively, and have the advantage of being very simple; however, an attempt was made to improve their predictive abilities by adding further independent variables. Equation (10) (Figure 5) contains, apart from (***N+O***), log ***D*** and ***R_M_***/***V_M_*** some more variables, ***M_W_*** and ***PSA***, traditionally linked to good absorption properties, and it accounts for over 91% of total variability:

log ***K_p_*** = −1.559 (±0.181) − 0.381 (±0.054) (***N+O***) + 0.176 (±0.036) log ***D*** + 146.43 (±37.66) ***R_M_***/***V_M_*** − 0.0000100 (±0.0000019) ***E_T_*** +0.0469 (±0.014) ***E_h_*** − 0.0095 (±0.0020) ***M_W_*** − 0.00048 (±0.00011) ***E_b_*** + 0.0108 (±0.0037) ***PSA*** (n = 60, R^2^ = 0.913, R^2^_adj._ = 0.899, F = 66.710, *p* < 0.0000, s_e_ = 0.332) (10)

The same set of variables as in Equation (10) was applied to the training set (compounds **1** to **40**). The Equation (11) generated at this stage was as follows:

log ***K_p_*** = −1.426 (±0.283) − 0.398 (±0.130) (***N+O***) + 0.157 (±0.063) log ***D*** + 168.41 (±51.99) ***R_M_***/***V_M_*** − 0.0000327 (±0.0000221) ***E_T_*** +0.0237 (±0.0204) ***E_h_*** − 0.0116 (±0.0046) ***M_W_*** − 0.000255 (±0.000189) ***E_b_*** + 0.00305 (±0.00813) ***PSA*** (*n* = 40, R^2^ = 0.863, R^2^_adj._ = 0.827, F = 24.361, *p* < 0.0000, s_e_ = 0.428) (11)

Equation (11) improved significantly after one compound (**19**) was removed. The difference between the ***K_p_***^(11)^ and ***K_p_***^EPI^ values for this compound is probably due to its very high energies and ***PSA,*** coincidentally leading to overestimated skin permeability, calculated according to Equation (11):

log ***K_p_*** = −1.590 (±0.185) − 0.399 (±0.084) (***N+O***) + 0.178 (±0.041) log ***D*** + 138.00 (±34.01) ***R_M_***/***V_M_*** − 0.000014 (±0.000015) ***E_T_*** +0.067 (±0.015) ***E_h_*** − 0.012 (±0.003) ***M_W_*** − 0.000568 (±0.000131) ***E_b_*** + 0.014 (±0.006) ***PSA*** (*n* = 39, R^2^ = 0.937, R^2^_adj._ = 0.921, F = 56.137, *p* < 0.0000, s_e_ = 0.278) (12)

The values of log ***K_p_*** were calculated for compounds **41** to **60** according to Equation (12) and plotted against the reference log ***K_p_***^EPI^ values. Just as in the case of Equation (9), the linear relationship between these two groups of log ***K_p_*** values improved when salicylic acid (**60**) was removed as an outlier (R^2^ = 0.831 and 0.886, respectively).

The model (10) was also tested on a subgroup of nine compounds, analyzed in this study whose log ***K_p_***^exp^ values were available (**12**, **16**, **21**, **40**, **56**, **60**, **61**, **62**, **63**). The resulting dependence was linear, with R^2^ = 0.83, which is a much better result than that obtained for the relationship between log ***K_p_***^exp^ and log ***K_p_***^EPI^.

## 3. Materials and Methods

### 3.1. Chemicals

The 63 drugs analyzed during these investigations were isolated from pharmaceutical preparations, purchased from Sigma-Aldrich (St. Louis, MO, USA)or donated as free samples by Polfa-Pabianice (Pabianice, Poland). The purity of drugs isolated from pharmaceutical preparations was assessed by TLC and densitometry (Section 3). All isolated drugs gave single chromatographic spots (densitometric peaks) and were used without further purification. Drugs purchased from Sigma-Aldrich were of analytical or pharmacopeial grade. Distilled water used for chromatography was from an in-house distillation apparatus. Analytical grade acetonitrile and methanol were from Avantor Performance Materials Poland S.A. (formerly POCh S.A., Gliwice, Poland). pH 7.4 phosphate buffered saline was from Sigma-Aldrich.

### 3.2. Thin Layer Chromatography

Thin layer chromatography was performed on 10 × 20 cm glass-backed RP-18 F_254s_ TLC plates (layer thickness 0.25 mm) from Merck (Darmstadt, Germany). Before use, the plates were pre-washed with methanol-dichloromethane 1:1 (*v*/*v*) and dried overnight in ambient conditions. Solutions of compounds **1** to **63** in methanol (1 μg·μL^−1^, spotting volume 1 μL), were spotted with a Hamilton microsyringe, 15 mm from the plate bottom edge, starting 10 mm from the plate edge, at 8 mm intervals. The chromatographic plates were developed in a vertical chromatographic chamber lined with filter paper and previously saturated with the mobile phase vapor for 20 min. The mobile phase consisted of acetonitrile—pH 7.4 phosphate buffered saline 70:30 (*v*/*v*) or methanol—pH 7.4 phosphate buffered saline (methanol contents from 90 to 50% *v*/*v* in 5% increments). The development distance was 95 mm from the plate bottom edge. After development, the plates were dried at room temperature and examined under UV light (254nm) and with a CD60 densitometer (Desaga, Germany, multiwavelength scan, 200–300 nm at 20 nm intervals). All chromatograms were repeated in duplicate, and the mean ***R_f_*** values were used in further investigations. The chromatographic data are presented in Table 4.

### 3.3. Calculated Molecular Descriptors

The molecular descriptors for compounds investigated during this study were calculated with HyperChem 8.0, utilizing PM3 semi-empirical method with the Polak-Ribiere algorithm: total dipole moment—***DM*** [D], logarithm of the octanol-water partition coefficient—log ***P***, van der Waals molar volume—***V_M_*** [Å^3^], surface area (grid and approximate)—***S_a_*** [Å^2^], molecular weight—***M_w_*** [g mol^−1^], energy of the highest occupied molecular orbital—***E_HOMO_*** [eV], energy of the lowest unoccupied molecular orbital—***E_LUMO_*** [eV], total energy—***E_T_*** [kcal mol^−1^], binding energy—***E_b_*** [kcal mol^−1^], electronic energy—***E_e_*** [kcal mol^−1^], hydration energy—***E_h_*** [kcal mol^−1^], refractivity—***R* [**Å^3^], polarizability—***α* [**Å^3^]. Other physicochemical parameters (distribution coefficient—log ***D***, polar surface area—***PSA* [**Å^2^], H-bond donor count—***HD,*** and H-bond acceptor count—***HA***) were calculated using ACD/Labs 8.0 software. The calculated molecular descriptors are given in Table 5. Statistical analysis was done using Statistica v.13 or StatistiXL v. 2.

## 4. Conclusions

This work has established that ***R_M_***/***V_M_*** is a useful descriptor of skin permeability derived from RP-18 thin layer chromatography. In a search for reliable log ***K_p_*** models based on this descriptor two possibilities were considered: a relatively simple model based on 5 independent variables: (***N+O***), log ***D***, ***R_M_***/***V_M_***, ***E_T_*** and ***E_h_*** (Equations (8) and (9)) and a more complex one, containing also ***E_b_***, ***M_W_*** and ***PSA*** (Equations (10)–(12)). The latter accounts for over 90% of total variability and involves all the major properties that determine the drugs’ ability to cross biological barriers (lipophilicity, molecular size, ability to form hydrogen bonds). It should be mentioned here that a very simple parameter (***N***+***O***) accounts for as much as 70% of the total log ***K_p_*** variability; log ***D*** and ***R_M_***/***V_M_*** account for a further 10 and 5%, respectively.

Skin permeability is a difficult property to measure.Due to the limited availability of experimental permeability data for the solutes investigated in this study, the reference skin permeability coefficients were calculated according to a widely accepted model based on log ***P_ow_*** and M_W_ (log ***K_p_***^EPI^).

The advantages of this model are clear—it is based on easily obtained molecular properties, whose influence upon the skin permeability is well documented [21]. Of course, the reference model has also its limitations: it overestimates the results for very hydrophilic molecules [52,53], underestimates the values for non-hydrogen bonding solutes [52], and fails for extremely lipophilic compounds or solutes having a very high tendency to hydrogen bonding [18,53]. However, the group of solutes examined in this study does not include molecules of very high lipophilicity or with a very high tendency to H-bonding, and the differences between log ***K_p_***^EPI^ and log ***K_p_***^exp^ for hydrophilic solutes or non-H-bond donors are moderate. Small discrepancies between the calculated and experimental reference values (log ***K_p_***^EPI^ and log ***K_p_***^exp^, respectively) for hydrophilic solutes or those without the H-donor sites may be a likely reason why the correlations between the experimental log ***K_p_***^exp^ values (where available) and the log *K_p_* values calculated according to the equations developed in this study ((6), (8) and (10)) are better than those between log ***K****_p_*^exp^ and log ***K****_p_*^EPI^ for the same subgroup of compounds.

To conclude, Equations (6), (8) and (10) have been shown to be efficient tools for skin permeability predictions. Although Equation (10) provides the closest correlation, Equations (6) and (8) have the advantages of clarity and avoidance of colinearity between the variables; the simplest solutions are usually the best.

## Figures and Tables

**Table 1:** Selected computational models of skin permeation.

Equation	*n*	*R*	Ref.
log ***k_p_*** = −1.36 Δlog ***P_oct_****_-**hept**_*− 3.38	21	0.90	[4]
log ***K_p_*** = −6.3 + 0.71 log ***P_ow_*** − 0.0061 ***M_w_***	93	0.82	[6]
log ***K_p_*** = 0.82 log ***P_ow_*** − 0.0093 ***V_M_*** − 0.039 ***M_Pt_*** − 2.36	60	0.95	[8]
log ***K_p_*** = 0.84 log ***P_ow_*** − 0.07 (log ***P_ow_***)^2^ − 0.27 ***H_b_*** − 1.84 log ***M_w_*** + 4.39	22	0.98	[23]
log ***K_p_*** = 0.652 log ***P_ow_*** − 0.00603 ***M_w_*** − 0.623 ***ABSQon*** − 0.313 ***SsssCH*** − 2.30	143	0.95	[13]
log ***K_p_*** = −5.426 − 0.106 ***E*** − 0.473 ***S*** − 0.473 ***A*** − 3.000 ***B*** + 2.296 ***V***	119	0.91	[12]
log ***K_p_*** = −3.05 − 0.0065 ***QXXp*** + 0.65 ***ALOGP*** − 1.75 ***Neoplastic-80*** + 0.22 ***F06****[**C-N**]*	158	0.91	[14]
log ***K_p_*** = −5.426 − 0.106 ***E*** − 0.473 ***S*** − 0.473 ***A*** − 3.000 ***B*** + 2.296 ***V***	119	0.91	[12]
log ***K_p_*** = −5.048 − 0.586 ***π*_2_*^H^*** − 0.633**Σ*α*_2_*^H^*** − 3.481 **Σ*β*_2_*^H^*** + 1.787 ***V***	46	0.98	[10]

Where: log ***P_ow_***—octanol-water partition coefficient; Δlog ***P_oct-hept_***—the difference between logarithms of octanol-water and heptane-water partition coefficients; ***M_W_***—molecular weight; ***V_M_***—molecular volume; ***M_Pt_***—melting point; ***SsssCH***—sum of E-state indices for all methyl groups; ***ABSQon***—sum of absolute charges on nitrogen and oxygen atoms; ***H_b_***—total H-bond count; ***A***, ***B***, ***S***, ***E***, ***V***—Abraham’s solvation parameters (***A***—hydrogen bond acidity; ***B***—hydrogen bond basicity; ***S***—dipolar interactions; ***E***—excess molar refractivity; ***V***—McGowan’s characteristic volume); ***Neoplastic-80***—antineoplastic-like property at 80% similarity; ***ALOGP***—log ***P_ow_*** calculated using ALOGP algorithm; ***F06[C-N]***—frequency of carbon-nitrogen bond at a topological distance of 06; ***QXXp***—electrostatic interactions between electric quadrupoles of van der Waals forces; ***π*_2_*^H^***—solute dipolarity/polarizability; Σ***α*_2_*^H^***—solute overall hydrogen-bond acidity; ***β*_2_*^H^***—solute overall hydrogen bond basicity.

**Table 2:** Selected relationships between skin permeability and liquid chromatographic descriptors.

	Equation	*n*	*R*	Reference
NP TLC	log ***K_p_*** = −1.318 (***R_M_***^0^)*^2^* − 7.529 ***R_M_***^0^ − 9.142 (dioxane-cyclohexane)	7 7	0.98	[30]
	log ***K_p_*** = −0.762 (***R_M_***^0^)^2^ − 5.146 ***R_M_***^0^ − 6.955 (THF-cyclohexane)		0.97	
IAM HPLC	log ***K_p_***= −10.19 + 1.77 log ***k^IAM^***	10	0.94	[31]
	log ***K_sc_***= 0.40 + 0.64 log ***k^IAM^***	10	0.97	
	log ***K_p_***= −6.16 − 0.46 (log ***k^IAM^***)^2^ + 1.54 log ***k^IAM^***	14	0.80	
	log ***K_p_*** = −6.09 + 1.05 log ***k^IAM^***	14	0.88	
	log ***K_p_***= −5.154 + 1.443 log ***k^IAM^***	32	0.51	[33]
	log ***K_sc_***= 1.555 + 1.522 log ***k^IAM^***	15	0.92	
	log ***K_p_***= −5.09 + 1.94 log ***k^IAM^***	32	0.55	[34]
	log ***K_p_***= −3.58 + 2.56 log ***k^IAM^*** − 1.12 *V*	32	0.86	
	log ***K_p_***= −2.419 Δlog ***k_w_^IAM^*** − 2.206	10	0.95	[32]
	log ***K_p_***= −2.136 Δlog ***k_w_^IAM^*** + 0.037 log ***P_ow_*** − 2.373	10	0.94	
	log ***K_p_***= −2.182 Δlog ***k_w_^IAM^*** + 0.046 log ***k_w_^IAM^*** − 2.323	10	0.94	
RP-18	log ***K_p_*** = −4.76 + 1.44 log ***k*** − 1.16 ***V***	27	0.91	[34]
	log ***K_p_*** = −5.728 + 1.636 log ***k*** (MSC18 column)	32	0.75	[33]
	log ***K_p_*** = −5.865 + 1.849 log ***k***(RP-18 column)	32	0.73	
	log ***K_sc_*** = 1.131 + 0.855 log ***k*** (MSC18 column)	15	0.87	
	log ***K_sc_*** = 1.099 + 0.95 log ***k*** (RP-18 column)	15	0.85	
BMC	log ***K_p_*** = −3.3 + 1.3 log ***k_BMC_*** − 0.008 ***M_Pt_***	42	0.91	[37]
	log ***K_p_*** = −2.24 + 1.83 log ***P_mw_*** − 0.0123 ***M_w_***	22	0.91	[39]
Keratin	log ***K_p_*** = −6.558 + 1.920 log ***k^IAM^*** − 1.039 log ***k_KERATIN_***	17	0.93	[36]

Where: ***K_p_***—skin permeability coefficient; ***R_f_***—retention factor in thin layer chromatography; ***R_M_*** = log (1/***R_f_*** − 1) and ***R_M_*^0^** are obtained by extrapolation of ***R_M_*** values from a series of TLC experiments conducted for different concentrations ***φ*** of a modifier in mobile phases to zero concentration of the modifier, according to the equation: ***R_M_*** = ***R_M_*^0^** + ***Sφ***; ***P_mw_***—micelle-water partitioning coefficient; ***k***—retention factor in column liquid chromatography; ***k^IAM^***, ***k_BMC_***, ***k****_KERATIN_*—retention factors in IAM, BMC and immobilized keratin chromatography, respectively; ***k_w_^IAM^***—***k^IAM^*** measured at or extrapolated to purely aqueous conditions; Δlog ***k_w_^IAM^***—the difference between log ***k_w_^IAM^*** measured and predicted on the basis of log ***P_ow_***; ***V***—McGowan’s characteristic volume; ***M_w_***—molecular weight; ***M_Pt_***—melting point; ***K_sc_***—human skin-water partition coefficient.

**Table 3:** Reference, experimental and calculated values of log ***K_p._***

		log *K_p_*^EPI^	log *K_p_*^(6)^	log *K_p_*^(7)^	log *K_p_*^(8)^	log *K_p_*^(9)^	log *K_p_*^(10)^	log *K_p_*^(12)^	log *K_p_*^exp^
**1**	Diazepam	−2.53	−1.99	−1.99	−1.94	−1.95	−2.02	−2.01	
**2**	Temazepam	−3.04	−2.68	−2.70	−2.63	−2.66	−2.77	−2.80	
**3**	Alprazolam	−3.13	−2.41	−2.39	−2.47	−2.43	−2.69	−2.82	
**4**	Medazepam	−1.39	−1.41	−1.41	−1.53	−1.54	−1.68	−1.67	
**5**	Bromazepam	−3.22	−2.71	−2.73	−2.64	−2.65	−3.17	−3.25	
**6**	Chlordiazepoxide	−2.87	−2.66	−2.68	−2.69	−2.73	−2.83	−2.84	
**7**	Midazolam	−1.75	−1.83	−1.82	−1.92	−1.97	−2.29	−2.30	
**8**	Oxazepam	−2.92	−2.90	−2.94	−2.70	−2.73	−2.80	−2.80	
**9**	Lorazepam	−3.02	−2.87	−2.91	−2.71	−2.74	−3.14	−3.22	
**10**	Lormetazepam	−3.20	−2.66	−2.68	−2.48	−2.53	−2.87	−2.87	
**11**	Clorazepate	−3.21	−3.16	−3.15	−2.95	−2.94	−2.94	−2.96	
**12**	Ibuprofen	−1.32	−1.74	−1.80	−1.69	−1.66	−1.23	−1.14	−1.44 [9]
**13**	Zolpidem	−1.97	−2.32	−2.29	−2.41	−2.47	−2.24	−2.10	
**14**	Tamoxifen	−0.70	−0.87	−0.80	−0.89	−0.89	−0.61	−0.51	
**15**	Propranolol	−1.95	−2.42	−2.48	−2.41	−2.46	−2.25	−2.18	
**16**	Ranitidine	−4.39	−4.12	−4.09	−4.62	−4.58	−4.62	−4.83	−4.05 [9]
**17**	Methyldopa	−5.18	−4.27	−4.41	−4.58	−4.70	−4.66	−4.67	
**18**	Amizepin	−2.50	−2.49	−2.56	−2.29	−2.29	−2.36	−2.38	
**19**	Enalapril	−4.87	−3.93	−3.88	−4.74	−3.65	−4.94	−2.55	
**20**	Paracetamol	−3.35	−2.85	−2.97	−3.27	−3.34	−3.40	−3.41	
**21**	Aspirin	−3.03	−2.84	−2.88	−3.17	−3.21	−3.07	−3.00	−2.14 [9]
**22**	Cefuroxime	−5.29	−6.45	−6.33	−6.00	−6.03	−5.56	−5.38	
**23**	Theophylline	−3.84	−3.76	−3.77	−3.86	−3.84	−3.69	−3.63	
**24**	Verapamil	−2.84	−3.14	−3.06	−3.14	−3.27	−2.93	−2.80	
**25**	Clobazam	−3.08	−2.55	−2.56	−2.58	−2.64	−2.81	−2.79	
**26**	Mitrazapin	−2.28	−2.14	−2.17	−2.22	−2.27	−2.17	−2.11	
**27**	Promazine	−1.38	−1.68	−1.73	−1.76	−1.82	−1.71	−1.62	
**28**	Phenytoin	−2.58	−2.87	−2.91	−2.69	−2.69	−2.50	−2.46	
**29**	Hydroxyzine	−3.34	−2.71	−2.74	−2.62	−2.72	−2.86	−2.89	
**30**	Mianserin	−1.47	−1.66	−1.71	−1.74	−1.80	−1.73	−1.67	
**31**	Valproic acid	−1.79	−2.30	−2.43	−2.27	−2.30	−1.90	−1.83	
**32**	Zopiclone	−3.97	−4.10	−4.00	−4.13	−4.21	−4.25	−4.14	
**33**	Haloperidol	−2.06	−2.35	−2.40	−2.08	−2.24	−2.28	−2.19	
**34**	Risperidone	−2.79	−3.15	−3.07	−3.07	−3.18	−2.94	−2.79	
**35**	Loperamide	−2.06	−2.48	−2.47	−2.14	−2.29	−2.21	−2.11	
**36**	Phenylbutazone	−2.44	−2.78	−2.82	−2.73	−2.84	−2.66	−2.57	
**37**	Clonidine	−3.04	−2.80	−2.91	−2.63	−2.68	−3.16	−3.25	
**38**	PABA	−3.02	−3.38	−3.57	−3.22	−3.26	−3.09	−3.11	
**39**	Propylparaben	−1.80	−2.23	−2.27	−2.35	−2.31	−2.06	−2.05	
**40**	Methylparaben	−2.36	−2.39	−2.45	−2.61	−2.57	−2.44	−2.47	−2.04 [8]
**41**	Quetiap Quetiapine	−3.67	−3.14	−3.14	−3.11	−3.19	−3.09	−3.07	
**42**	Chlorprotixen	−1.13	−1.06	−1.10	−1.09	−1.14	−1.25	−1.20	
**43**	Perazine	−2.05	−1.96	−1.96	−1.93	−1.99	−1.84	−1.74	
**44**	Trifluoperazine	−1.75	−1.79	−1.77	−1.58	−1.73	−1.79	−1.65	
**45**	Thioridazine	−0.96	−1.48	−1.50	−1.44	−1.52	−1.27	−1.09	
**46**	Fluconazole	−4.19	−4.00	−3.96	−4.00	−4.04	−4.21	−4.24	
**47**	Tolperisone	−1.76	−1.74	−1.80	−1.75	−1.82	−1.45	−1.28	
**48**	Fenspiride	−2.75	−3.02	−3.09	−2.91	−3.00	−2.75	−2.66	
**49**	Pizotifen	−1.00	−1.04	−1.09	−1.08	−1.12	−0.87	−0.75	
**50**	Cyproheptadine	−1.30	−0.99	−1.02	−1.05	−1.08	−0.93	−0.86	
**51**	Clozapine	−2.52	−2.91	−2.96	−2.69	−2.79	−2.99	−2.97	
**52**	Tiapride	−4.05	−3.96	−3.99	−3.77	−3.91	−3.66	−3.51	
**53**	Olanzapine	−2.56	−2.61	−2.62	−2.50	−2.52	−2.34	−2.25	
**54**	Betahistine	−3.12	−2.66	−2.84	−2.68	−2.75	−2.66	−2.66	
**55**	Dexketoprofen	−2.16	−2.71	−2.82	−2.61	−2.70	−2.45	−2.37	
**56**	Caffeine	−3.94	−3.52	−3.50	−3.68	−3.68	−3.58	−3.51	−3.64 [34]
**57**	Hymecromone	−2.53	−2.66	−2.68	−2.89	−2.84	−2.77	−2.83	
**58**	Ketotifen	−1.99	−1.58	−1.62	−1.64	−1.71	−1.47	−1.32	
**59**	Clemastine	−1.09	−1.62	−1.66	−1.52	−1.63	−1.64	−1.57	
**60**	Salicylic acid	−2.08	−3.07	−3.22	−3.09	−3.10	−2.99	−3.05	−2.84 [9]
**61**	Indomethacin	−1.97	−3.40	−3.44	−3.24	−3.36	−3.49	−3.51	−3.67 [9,13]
**62**	Piroxicam	−2.63	−4.05	−4.00	−3.85	−3.84	−3.68	−3.65	−3.81 [9,13]
**63**	Naproxen	−1.98	−2.60	−2.69	−2.63	−2.68	−2.52	−2.54	−2.54 [37]

Where: log ***K_p_***^exp^—experimental values; log ***K_p_***^EPI^—values calculated using DERMWIN software; log ***K_p_***^(6)^ to log ***K_p_***^(10)^ and log ***K_p_***^(12)^—values calculated according to Equations (6)–(10) and (12).

**Table 4:** Chromatographic parameters for compounds **1** to **63**.

		*R_f_*	*R_f_*/*PSA*	*R_M_*	*R_M_*/*M_W_*	*R_M_*/*V_M_*	*R_M_* ^0^	*S*
**1**	Diazepam	0.33	0.0101	0.308	0.00108	0.00123	3.578	−4.176
**2**	Temazepam	0.50	0.0095	0.000	0.00000	0.00000	3.050	−3.798
**3**	Alprazolam	0.22	0.0058	0.550	0.00178	0.00204	3.254	−3.877
**4**	Medazepam	0.56	0.0359	−0.105	−0.00039	−0.00042	2.528	−3.035
**5**	Bromazepam	0.51	0.0094	−0.017	−0.00005	−0.00007	2.496	−3.269
**6**	Chlordiazepoxide	0.62	0.0117	−0.213	−0.00070	−0.00079	2.131	−2.551
**7**	Midazolam	0.58	0.0230	−0.140	−0.00043	−0.00051	2.235	−2.741
**8**	Oxazepam	0.60	0.0097	−0.176	−0.00061	−0.00073	2.864	−3.752
**9**	Lorazepam	0.61	0.0099	−0.194	−0.00060	−0.00076	3.031	−4.037
**10**	Lormetazepam	0.49	0.0093	0.017	0.00005	0.00006	3.303	−4.207
**11**	Clorazepate	0.47	0.0060	0.052	0.00017	0.00020	3.117	−3.840
**12**	Ibuprofen	0.46	0.0122	0.078	0.00038	0.00037	4.139	−4.895
**13**	Zolpidem	0.68	0.0181	−0.327	−0.00106	−0.00109	1.744	−2.341
**14**	Tamoxifen	0.48	0.0381	0.043	0.00012	0.00015	5.041	−5.908
**15**	Propranolol	0.82	0.0198	−0.659	−0.00254	−0.00254	2.059	−3.004
**16**	Ranitidine	0.82	0.0074	−0.659	−0.00208	−0.00220	0.343	−0.518
**17**	Methyldopa	0.98	0.0094	−1.690	−0.00800	−0.00914	2.480	−0.197
**18**	Amizepin	0.55	0.0119	−0.087	−0.00033	−0.00040	2.484	−3.221
**19**	Enalapril	0.82	0.0085	−0.659	−0.00175	−0.00175	2.035	−2.994
**20**	Paracetamol	0.85	0.0172	−0.753	−0.00498	−0.00542	0.459	−1.500
**21**	Aspirin	0.76	0.0119	−0.501	−0.00278	−0.00328	1.301	−2.204
**22**	Cefuroxime	0.77	0.0039	−0.525	−0.00124	−0.00160	2.023	−3.307
**23**	Theophylline	0.79	0.0114	−0.575	−0.00319	−0.00110		
**24**	Verapamil	0.77	0.0120	−0.525	−0.00115	−0.00036		
**25**	Clobazam	0.50	0.0124	0	0	0		
**26**	Mitrazapin	0.71	0.0366	−0.389	−0.00147	−0.00049		
**27**	Promazine	0.71	0.0223	−0.389	−0.00137	−0.00045		
**28**	Phenytoin	0.70	0.0120	−0.368	−0.00146	−0.00051		
**29**	Hydroxyzine	0.76	0.0212	−0.501	−0.00134	−0.00046		
**30**	Mianserin	0.77	0.1185	−0.525	−0.00199	−0.00065		
**31**	Valproic acid	0.54	0.1185	−0.070	−0.00048	−0.00013		
**32**	Zopiclone	0.80	0.1185	−0.602	−0.00155	−0.00059		
**33**	Haloperidol	0.78	0.0192	−0.550	−0.00146	−0.00053		
**34**	Risperidone	0.55	0.0089	−0.087	−0.00021	−0.00008		
**35**	Loperamide	0.53	0.0121	−0.052	−0.00011	−0.00004		
**36**	Phenylbutazone	0.39	0.0096	0.194	0.00063	0.00021		
**37**	Clonidine	0.83	0.0234	−0.689	−0.00299	−0.00110		
**38**	PABA	0.85	0.0134	−0.753	−0.00549	−0.00170		
**39**	Propylparaben	0.61	0.0131	−0.194	−0.00108	−0.00032		
**40**	Methylparaben	0.72	0.0155	−0.410	−0.00270	−0.00084		
**41**	Quetiapine	0.72	0.0098	−0.410	−0.00107	−0.00037		
**42**	Chlorprotixen	0.57	0.0200	−0.122	−0.00039	−0.00013		
**43**	Perazine	0.44	0.0126	0.105	0.00031	0.00011		
**44**	Trifluoperazine	0.44	0.0126	0.105	0.00026	0.00010		
**45**	Thioridazine	0.52	0.0091	−0.035	−0.00009	−0.00003		
**46**	Fluconazole	0.68	0.0095	−0.327	−0.00107	−0.00042		
**47**	Tolperisone	0.75	0.0369	−0.477	−0.00194	−0.00059		
**48**	Fenspiride	0.80	0.0178	−0.602	−0.00231	−0.00076		
**49**	Pizotifen	0.55	0.0175	−0.087	−0.00029	−0.00010		
**50**	Cyproheptadine	0.62	0.1914	−0.213	−0.00074	−0.00024		
**51**	Clozapine	0.68	0.0220	−0.327	−0.00100	−0.00035		
**52**	Tiapride	0.81	0.0096	−0.630	−0.00192	−0.00064		
**53**	Olanzapine	0.60	0.0102	−0.176	−0.00056	−0.00020		
**54**	Betahistine	0.72	0.0289	−0.410	−0.00301	−0.00079		
**55**	Dexketoprofen	0.61	0.0112	−0.194	−0.00076	−0.00025		
**56**	Caffeine	0.66	0.0123	−0.288	−0.00148	−0.00050		
**57**	Hymecromone	0.73	0.0157	−0.432	−0.00245	−0.00083		
**58**	Ketotifen	0.70	0.0144	−0.368	−0.00119	−0.00042		
**59**	Clemastine	0.45	0.0361	0.087	0.00025	0.00009		
**60**	Salicylic acid	0.70	0.0122	−0.368	−0.00266	−0.00088		
**61**	Indomethacin	0.46	0.0066	−0.069	0.000195	0.00007		
**62**	Piroxicam	0.50	0.0046	0	0	0	2.598	−3.193
**63**	Naproxen	0.59	0.0127	−0.158	−0.00069	−0.00022		

**Table 5:** Calculated descriptors for compounds ***1*** to ***63***.

		*PSA*	*HD*	*HA*	log *D*	*N+O*	*M_W_*	*E_T_*	*E_b_*	*E_e_*	*DM*	*E_HOMO_*	*E_LUMO_*	*S_a_(a)*	*S_a_(g)*	*V_M_*	*E_h_*	log *P*	*R*	*α*
**1**	Diazepam	32.7	0	3	2.96	3	284.7	−69,868	−3697	−488,334	3.32	−9.20	−0.76	279.6	283.8	250.5	−2.75	0.94	87.8	31.0
**2**	Temazepam	52.9	1	4	2.20	4	300.7	−76,642	−3799	−541,464	3.98	−9.27	−0.87	289.2	291.9	257.1	−7.84	1.08	89.0	31.6
**3**	Alprazolam	38.1	0	4	2.50	4	308.8	−73,284	−3950	−550,378	6.48	−9.65	−1.23	293.7	299.4	269.4	−9.24	2.13	95.4	33.8
**4**	Medazepam	15.6	0	2	4.43	2	270.8	−63,807	−3705	−454,146	2.35	−8.82	−0.40	277.4	284.4	250.0	−1.58	1.75	88.1	30.9
**5**	Bromazepam	54.4	1	4	2.06	4	316.2	−67,922	−3290	−441,623	3.46	−9.33	−0.79	255.3	270.6	236.5	−5.68	−0.42	83.4	29.1
**6**	Chlordiazepoxide	53.1	1	4	2.36	4	301.8	−74,644	−3933	−566,228	1.88	−8.67	−0.31	304.6	304.2	268.3	−7.93	0.39	91.5	32.9
**7**	Midazolam	25.3	0	3	3.93	3	325.8	−82,444	−4091	−609,612	4.38	−9.17	−0.96	300.0	309.6	277.3	−3.37	0.13	98.4	34.4
**8**	Oxazepam	61.7	2	4	2.31	4	286.7	−73,199	−3524	−494,470	2.74	−9.13	−0.73	266.5	274.9	240.7	−7.92	0.84	84.1	29.8
**9**	Lorazepam	61.7	2	4	2.47	4	321.2	−80,151	−3508	−544,373	4.02	−9.28	−0.87	283.6	291.3	254.6	−9.96	0.61	88.8	31.7
**10**	Lormetazepam	52.9	1	4	2.36	4	335.2	−83,588	−3778	−599,987	1.93	−9.12	−0.64	307.2	308.3	271.7	−4.85	0.86	93.7	33.5
**11**	Clorazepate	78.8	2	5	2.90	5	314.7	−82,710	−3797	−581,135	3.32	−9.27	−0.93	288.7	293.6	258.0	−10.98	0.68	88.9	31.7
**12**	Ibuprofen	37.3	1	2	3.72	2	206.3	−55,498	−3380	−344,748	1.86	−9.51	0.06	262.5	251.3	210.1	−4.81	2.75	64.1	24.0
**13**	Zolpidem	37.6	0	4	3.07	4	307.4	−77,368	−4724	−613,528	4.07	−8.56	−0.55	355.6	351.1	300.7	−1.03	−0.22	99.6	35.9
**14**	Tamoxifen	12.5	0	2	7.88	2	371.5	−91,827	−6085	−810,401	0.59	−8.88	0.09	446.1	438.0	283.3	−2.55	2.88	131.6	46.2
**15**	Propranolol	41.5	2	3	3.10	3	259.4	−68,465	−4113	−472,868	1.26	−8.62	−0.43	311.9	307.7	259.4	−7.41	0.68	83.4	30.3
**16**	Ranitidine	111.6	2	7	1.23	7	316.4	−83,560	−4192	−562,535	3.41	−7.96	−0.21	403.6	378.1	299.3	−28.08	−4.02	89.1	33.6
**17**	Methyldopa	103.8	5	5	0.12	5	211.2	−62,797	−2902	−363,522	2.59	−9.03	−0.01	225.9	224.7	185.0	−21.50	−1.46	57.2	21.1
**18**	Amizepin	46.3	2	3	2.67	3	263.3	−59,477	−3441	−409,257	3.32	−9.01	−0.59	243.5	249.9	219.2	−6.48	−0.28	80.0	27.4
**19**	Enalapril	95.9	2	7	2.43	7	376.5	−102,647	−5843	−879,009	2.57	−8.94	0.28	421.3	441.2	376.5	−2.24	2.57	111.0	42.3
**20**	Paracetamol	49.3	2	3	0.34	3	151.2	−42,346	−2132	−207,000	4.31	−8.43	0.21	174.8	172.1	139.0	−10.61	−1.32	45.6	16.2
**21**	Aspirin	63.6	1	4	1.19	4	180.2	−54,523	−2335	−277,770	0.49	−9.66	−0.57	192.6	187.3	152.8	−7.43	−0.26	48.0	17.4
**22**	Cefuroxime	199.1	4	12	0.47	12	424.4	−122,720	−4706	−1,017,047	3.74	−9.47	−1.61	222.3	377.3	328.4	−18.43	−2.18	99.7	38.1
**23**	Theophylline	69.3	1	6	−0.20	6	180.2	−50,484	−2233	−281,145	3.47	−9.11	−0.57	301.7	335.5	523.0	−5.34	−1.31	45.1	17.0
**24**	Verapamil	64.0	0	6	2.33	6	454.6	−121,717	−7044	−1,150,691	3.64	−8.78	−0.95	705.7	817.4	1453.9	−8.35	2.81	136.6	51.5
**25**	Clobazam	40.6	0	4	1.59	4	300.7	−76,643	−3800	−546,998	0.55	−8.68	−0.23	402.3	486.0	813.3	−3.41	−1.40	89.7	31.5
**26**	Mitrazapin	19.4	0	3	1.97	3	265.4	−64,413	−4174	−512,476	0.83	−8.38	0.08	352.3	476.4	798.4	−1.47	1.16	87.1	31.6
**27**	Promazine	31.8	0	2	2.63	2	284.4	−65,321	−4185	501,582	2.89	−7.84	−0.24	461.7	508.8	860.3	−1.65	1.37	93.7	34.2
**28**	Phenytoin	58.2	2	4	2.48	4	252.3	−66,250	−3542	−454,910	2.86	−9.90	−0.39	333.5	435.7	716.5	−7.67	2.26	70.0	27.7
**29**	Hydroxyzine	35.9	1	4	2.00	4	374.9	−95,297	−5407	805,537	1.70	−9.13	−0.11	563.4	637.5	1092.8	−8.90	3.49	107.1	41.7
**30**	Mianserin	6.5	0	2	2.76	2	264.4	−63,761	−4290	−510,168	0.77	−8.46	0.20	365.6	479.6	808.8	−0.80	0.94	91.2	32.3
**31**	Valproic acid	37.3	1	2	0.16	2	144.2	−41,139	−2450	−216,934	4.47	−11.03	1.11	372.6	356.7	539.8	−4.39	2.61	40.3	16.2
**32**	Zopiclone	91.8	0	9	0.65	8	388.8	−102,536	−4710	−838,854	4.51	−9.36	−1.28	489.2	598.1	1025.2	−5.05	−1.83	101.9	37.9
**33**	Haloperidol	40.5	1	3	2.11	3	375.9	−100,303	−5164	−774,341	1.14	−9.12	−0.70	526.5	605.2	1035.8	−4.30	3.38	102.6	39.8
**34**	Risperidone	61.9	0	6	2.27	6	410.5	−111,074	−5964	−985,849	5.49	−8.87	−0.88	489.7	640.6	1127.2	−4.39	0.63	118.5	43.5
**35**	Loperamide	43.8	1	4	3.53	4	477.0	−119,284	−7065	−1,205,052	3.73	−8.93	−0.09	588.1	713.8	1308.0	−5.22	5.01	139.4	54.5
**36**	Phenylbutazone	40.6	0	4	0.10	4	308.4	−80,026	−4646	−632,151	0.90	−9.32	−0.26	473.7	543.9	920.5	−3.25	1.84	98.4	35.0
**37**	Clonidine	35.4	2	3	0.65	3	230.1	−53,612	−2372	−304,330	1.17	−8.96	−0.19	321.3	401.1	624.9	−5.73	0.28	62.2	22.7
**38**	PABA	63.3	3	3	−1.61	3	137.1	−38,905	−1859	−176,902	4.29	−8.50	−0.21	247.8	298.6	442.5	−11.00	0.96	37.5	14.3
**39**	Propylparaben	46.5	1	3	2.87	3	180.2	−51,913	−2627	−272,388	1.43	−9.52	−0.40	378.1	386.8	599.2	−7.99	2.30	48.6	19.1
**40**	Methylparaben	46.5	1	3	1.81	3	152.2	−45,017	−2067	−210,788	1.54	−9.53	−0.42	308.2	325.8	488.0	−9.09	1.49	39.3	15.5
**41**	Quetiapine	73.6	1	5	1.55	5	383.5	−95,297	−5413	−823,576	2.20	−8.64	−0.70	535.3	637.5	1098.9	−11.29	2.84	111.6	43.0
**42**	Chlorprotixen	28.5	0	1	4.40	1	315.9	−70,896	−4164	−520,729	1.94	−8.35	−0.56	510.5	545.8	912.1	−1.47	4.33	95.5	36.4
**43**	Perazine	35.0	0	3	3.13	3	339.5	−79,041	−5068	−693,691	2.18	−7.81	−0.17	453.1	550.9	986.5	−1.37	−0.76	114.4	40.3
**44**	Trifluoperazine	35.0	0	3	4.21	3	407.5	−111,907	−5402	−911,148	3.51	−8.19	−0.79	534.0	599.0	1067.0	−0.91	−0.19	119.7	41.8
**45**	Thioridazine	57.1	0	2	3.94	2	370.6	−82,689	−5253	−744,945	0.86	−7.78	−0.41	484.9	581.1	1039.3	−1.39	−0.42	123.0	43.8
**46**	Fluconazole	71.8	1	7	0.50	7	306.3	−89,231	−3607	−625,777	0.86	−10.43	−1.03	345.7	469.7	783.0	−10.28	−1.41	79.5	28.6
**47**	Tolperisone	20.3	0	2	2.27	2	245.4	−62,429	−4147	−464,977	2.97	−9.05	−0.15	436.5	486.1	815.0	1.41	3.01	79.6	29.5
**48**	Fenspiride	45.1	1	4	0.04	4	260.3	−69,140	−4020	−499,628	5.09	−9.28	0.16	401.1	475.0	791.6	−4.01	1.40	73.7	28.8
**49**	Pizotifen	31.5	0	1	4.49	1	295.4	−67,399	−4466	−549,282	1.15	−8.89	−0.32	379.1	505.1	869.1	−0.98	1.58	95.7	35.7
**50**	Cyproheptadine	3.2	0	1	4.86	1	287.4	−68,558	−4726	−566,080	1.04	−8.73	−0.36	378.9	516.5	887.5	−1.13	1.77	102.8	36.0
**51**	Closapine	30.9	1	4	0.76	4	326.8	−78,188	−4481	−629,966	3.11	−8.45	−0.61	433.0	545.3	926.1	−2.78	−0.73	103.5	36.5
**52**	Tiapride	84.1	1	6	−1.48	6	328.4	−88,376	−4474	−658,862	5.32	−9.27	−0.73	605.9	582.1	979.5	−5.66	−1.56	91.2	31.4
**53**	Olanzapine	59.1	1	4	2.68	4	312.4	−72,794	−4389	−594,188	3.31	−8.21	−0.72	437.2	534.2	901.5	−4.10	1.66	95.3	35.9
**54**	Betahistine	24.9	1	2	−2.18	2	136.2	−33,605	−2197	−176,624	2.59	−9.19	−0.05	328.2	351.2	521.3	−4.27	−0.52	46.0	16.6
**55**	Dexketoprofen	54.4	1	3	−0.25	3	254.3	−69,002	−3723	−449,051	1.60	−9.97	−0.57	402.6	470.1	768.0	−6.46	2.56	79.9	28.2
**56**	Caffeine	53.5	0	6	−0.13	6	194.2	−53,927	−2508	−319,054	3.78	−8.90	−0.49	341.4	365.3	572.9	−2.21	−1.06	50.0	18.9
**57**	Hymecromone	46.5	1	3	2.36	4	176.2	−50,477	−2397	−262,408	5.98	−9.21	−0.91	289.5	341.9	520.9	−9.80	−0.56	51.5	18.2
**58**	Ketotifen	48.6	0	2	3.28	2	309.4	−73,450	−4448	−586,056	4.06	−9.09	−0.97	389.1	508.1	876.7	−2.17	0.26	99.6	35.8
**59**	Clemastine	12.5	0	2	3.04	2	343.9	−84,433	−5152	−711,749	1.96	−8.95	−0.15	501.2	584.3	1012.5	−0.80	4.66	101.4	39.6
**60**	Salicylic acid	57.5	2	3	−1.06	3	138.1	−41,583	−1801	−184,688	0.99	−9.45	−0.60	236.5	284.1	420.5	−11.88	−0.04	38.6	13.7
**61**	Indomethacin	69.6	1	5	−0.16	5	357.8	−95,725	−4971	−704,963	2.36	−8.56	−0.61	509.6	562.9	961.3	−9.47	−1.43	103.3	36.7
**62**	Piroxicam	108.0	2	7	1.71	7	331.3	−88,167	−3947	−657,874	5.06	−8.99	−1.21	320.8	308.3	268.4	−11.67	−2.25	91.6	30.3
**63**	Naproxen	46.5	1	3	0.47	3	230.3	−63,546	−3396	−390,177	2.42	−8.67	−0.53	395.2	445.4	703.3	−9.53	0.56	70.6	25.3

## Data Availability

The data presented in this study are available in this manuscript.

## Abbreviations

***K_p_***—skin permeability coefficient; ***PSA***—polar surface area; ***D***—distribution coefficient; ***P_ow_***—octanol-water partition coefficient; ***M_w_***—molecular weight; ***M_Pt_***—melting point; ***H_b_***—total H-bond count; ***SsssCH***—sum of E-state indices for all methyl groups; ***ABSQon***—sum of absolute charges on nitrogen and oxygen atoms; ***A***—hydrogen bond acidity; ***B***—hydrogen bond basicity; ***S***—dipolar interactions; ***E***—excess molar refractivity; ***V***—McGowan’s characteristic volume; ***H_d_***—H-bond donor activity; ***H_a_***—H-bond acceptor activity; IAM—immobilized artificial membrane; BMC—biomedical chromatography; ***k***—retention factor in column liquid chromatography; ***R_f_***—retention factor in TLC; TLC—thin layer chromatography; RP—reversed phase (chromatography); ***P_mw_***—micelle-water partitioning coefficient; ***k^IAM^*** and ***k_BMC_***—retention factors in IAM and BMC chromatography, respectively; log ***k_w_^IAM^***—log ***k^IAM^*** measured at or extrapolated to purely aqueous conditions; Δlog ***k_w_^IAM^***—the difference between log ***k_w_^IAM^*** measured and predicted on the basis of log ***P_ow_***; ***V***—McGowan’s characteristic volume; ***K_sc_***—human skin-water partition coefficient; ***DM***—total dipole moment; ***V_M_***—van der Waals molar volume; ***S_a_***_(a)_—surface area (approximate); ***S_a_***_(g)_ –surface area (grid); ***E_HOMO_***—energy of the highest occupied molecular; ***E_LUMO_***—energy of the lowest unoccupied molecular orbital; ***E_T_***—total energy; ***E_b_***—binding energy; ***E_e_***—electronic energy; ***E_h_***—hydration energy; ***R***—refractivity; ***α***—polarizability; ***HD***—H-bond donor count; ***HA***—H-bond acceptor count; (***N+O***)—total nitrogen and oxygen atom count; ***Neoplastic-80***—antineoplastic-like property at 80% similarity; ***ALOGP***—log ***P_ow_*** calculated using ALOGP algorithm; ***F06[C-N]***—frequency of carbon-nitrogen bond at a topological distance of 06; ***QXXp***—electrostatic interactions between electric quadrupoles of van der Waals forces; ***π*_2_*^H^***—solute dipolarity/polarizability; Σ***α*_2_*^H^***—solute overall hydrogen-bond acidity; ***β*_2_*^H^***—solute overall hydrogen bond basicity.
